# Stage-Specific Plasma Metabolomic Profiles in Colorectal Cancer

**DOI:** 10.3390/jcm13175202

**Published:** 2024-09-02

**Authors:** Tetsuo Ishizaki, Masahiro Sugimoto, Yu Kuboyama, Junichi Mazaki, Kenta Kasahara, Tomoya Tago, Ryutaro Udo, Kenichi Iwasaki, Yutaka Hayashi, Yuichi Nagakawa

**Affiliations:** 1 Department of Gastrointestinal and Pediatric Surgery, Tokyo Medical University, 6-7-1 Nishi-Shinjuku, Shinjuku 160-0023, Tokyo, Japan; tetsuo-i@tokyo-med.ac.jp (T.I.); kuboyama@tokyo-med.ac.jp (Y.K.); junichim@tokyo-med.ac.jp (J.M.); kasadog@tokyo-med.ac.jp (K.K.); t-tago@tokyo-med.ac.jp (T.T.); udo88@tokyo-med.ac.jp (R.U.); kiwasaki@tokyo-med.ac.jp (K.I.); rimpoo@tokyo-med.ac.jp (Y.H.); naga@tokyo-med.ac.jp (Y.N.); 2 Institute of Medical Science, Tokyo Medical University, Shinjuku 160-8402, Tokyo, Japan; 3 Institute for Advanced Biosciences, Keio University, Tsuruoka 997-0052, Yamagata, Japan

**Keywords:** colorectal cancer, metastasis, metabolomics, capillary electrophoresis–mass spectrometry, liquid chromatography–mass spectrometry, mass spectrometry

## Abstract

**Background/Objectives**: The objective of this study was to investigate the metabolomic profiles of patients with colorectal cancer (CRC) across various stages of the disease. **Methods**: The plasma samples were obtained from 255 subjects, including patients with CRC in stages I-IV, polyps, and controls. We employed capillary electrophoresis time-of-flight mass spectrometry and liquid chromatography triple quadrupole mass spectrometry to analyze hydrophilic metabolites comprehensively. The data were randomly divided into two groups, and consistent differences observed in both groups were analyzed. **Results**: Acetylated polyamines, such as *N*^1^-acetylspermine and *N*^1^, *N*^12^-diacetylspermine, consistently showed elevated concentrations in stage IV compared to stages I-III. Non-acetylated polyamines, including spermine and spermidine, exhibited increasing trends from polyp to stage IV. Other metabolites, such as histidine and o-acetylcarnitine, showed decreasing trends across stages. While acetylated polyamines have been reported as CRC detection markers, our findings suggest that they also possess diagnostic potential for distinguishing stage IV from other stages. **Conclusions**: This study showed stage-specific changes in metabolic profiles, including polyamines, of colorectal cancer.

## 1. Introduction

Colorectal cancer (CRC) is the most prevalent malignancy of the digestive system worldwide [1]. It ranks as the third most frequently diagnosed cancer in men and the second in women globally [1]. The majority of CRC-related deaths are due to metastatic disease, with 22% of such patients presenting with metastasis at the time of diagnosis [2]. The 5-year survival rate for stage I and II CRC is relatively high, ranging from 87 to 90%, whereas for stage III, it falls to 68–72%. For stage IV metastatic CRC, the survival rate is markedly lower, ranging from 11 to 14% [3].

The increase in CRC-diagnosed cases can be attributed to advances in imaging modalities such as computed tomography (CT), magnetic resonance imaging (MRI), and positron emission tomography (PET-CT), which have improved diagnostic accuracy and interpretation [4]. In addition, advances in diagnostic techniques that combine tumor markers, such as carcinoembryonic antigen (CEA) and carbohydrate antigen 19-9 (CA19-9), improve the ability to diagnose stage IV CRC [5]. The combination of these tumor markers was an effective predictor of prognosis in patients with stage IV CRC [6]. For stage IV CRC, which has a poorer prognosis compared to stages I–III, early multidisciplinary treatment, including surgery, chemotherapy, and radiotherapy, is essential. Research into compounds that can improve diagnostic accuracy and serve as a key to the treatment strategy for stage IV CRC is critical.

Liquid biopsy has recently gained attention as the antonym of solid biopsy [7]. Advances in the analysis techniques for minute specimens now allow for the analysis of materials such as cells and nucleic acids obtained from blood or bodily fluids in a minimally invasive manner, enabling a better understanding of disease pathophysiology. The advantages of liquid biopsies lie in their non-invasive nature, allowing for repeated testing without burdening the patient. In practice, liquid biopsies target various components, including circulating tumor cells in the bloodstream, tumor-derived DNA and RNA, proteins, and extracellular vesicles originating from tumor tissues [8,9,10,11]. Thus, the non-invasive nature of liquid biopsies has the potential to provide insights into disease detection, prognosis, and monitoring treatment outcomes [12].

Comprehensive molecular profiling techniques, or omics, have advanced in cancer biomarker discoveries. Metabolomics, which enables the profiling of small organic molecules called metabolites, has been used for colon cancer biomarker discovery. Several studies have been reported evaluating the different patterns of metabolites in the blood of CRC patients and healthy individuals [13,14]. A genome-wide association study and a large-scale metabolomic analysis of plasma and urine identified metabolites that are risk factors for CRC [15]. The metabolomic analysis of serum revealed CRC with adenomas and alterations in metabolites of lysolipids, glycerophosphocholine, acylcarnitine, and the arginine–urea cycle [16]. Abscisic acid, calcitroic acid, and glucosyl sphingosine were identified as substances showing significant differences in the presence or absence of lymph nodes in CRC [17]. We also analyzed metabolomics using saliva and urine samples collected from CRC patients and found that polyamines showed diagnostic potential [18,19,20,21]. Many of these studies aimed to search for markers to discriminate colorectal cancer from healthy individuals or develop models to discriminate using patterns of multiple metabolites.

This study aims to understand the relationship between plasma metabolites and the progress of CRC. Capillary electrophoresis–mass spectrometry (CE-MS) and liquid chromatography–MS (LC-MS) were used to profile hydrophilic metabolites, including polyamines. The metabolomics concentration of stages I-III and IV were compared to find metabolites with discrimination abilities. Stage-specific metabolomic profiles were also analyzed.

## 2. Materials and Methods

### 2.1. Study Design

This study was conducted following the principles of the Helsinki Declaration. The research protocol received approval from the Ethics Committee of Tokyo Medical University (Approval number: SH3915). Before participation in this study, informed consent was obtained from each participant through written documentation.

The study included patients diagnosed with pathological CRC as adenocarcinoma at Tokyo Medical University Hospital between January 2014 and December 2016. CRC was defined according to the following presentations: in the cecum, ascending colon, transverse colon, descending colon, sigmoid colon, rectosigmoid colon, and rectum. Stages I–III and stage IV were classified according to the 7th edition of the International Union Against Cancer (UICC) TNM classification. Patients with colitis-associated cancer and chronic metabolic diseases such as diabetes were also excluded. Blood samples (2 mL) were collected from the veins of fasting patients diagnosed with CRC between 7:00 and 8:00 am the day before medical intervention. Immediately, plasma samples were prepared from blood samples using EDTA-2Na+ and stored at −80 °C. Plasma samples were also collected from healthy controls and polyps.

### 2.2. Sample Preparation for Metabolome Analysis

For metabolome analysis of plasma samples, we employed CE–time of flight (TOF)MS for most metabolites and LC-(triple quadropole)QQQMS for polyamines. The sample preparation for CE-TOFMS was previously reported [22]. Thawed plasma samples were mixed with 360 μL of methanol and methionine sulfone, 20 μM each, and 20 μM of 2-(N-morpholino)ethanesulfonic acid and D-camphor-10-sulfonic acid. Subsequently, 160 μL of deionized water and 400 μL of chloroform were added, followed by centrifugation at 10,000× *g* for 3 min at 4 °C. The upper aqueous layer was filtered through a 5 kDa cutoff filter (Millipore, Billerica, MA, USA) at 9100× *g* for 180 min at 4 °C to remove large molecules. The remaining solution was concentrated by centrifugation (960× *g*) at 40 °C for 3 h and freeze-dried to the extent necessary for CE-TOFMS analysis. For metabolite analysis, the sample was dissolved in 40 μL of Milli-Q water containing 200 μM of 3-aminopyrrolidine and trimethylsulfonic acid for CE-TOFMS.

Sample processing for LC-QQQMS was performed as follows. Plasma (30 μL) was mixed with methanol (270 μL) containing 149.6 mM of ammonium hydroxide (1% (*v*/*v*) ammonia solution) and 0.2 μM of internal standards (d8-spermine, d8-spermidine, d6-*N*^1^-acetylspermidine, 1,6-diaminohexane, d6-*N*^1^,*N*^8^-diacetylspermidine, and d6-*N*^1^,*N*^12^-diacetylspermine). After centrifugation at 20,400× *g* for 10 min at 4 °C, the supernatant was transferred to another tube and vacuum-dried for 1 h and 40 min at 40 °C. The sample was reconstituted with 90% methanol (10 μL) and water (20 μL), vortexed, and then centrifuged at 6800× *g* for 5 min at 4 °C.

### 2.3. CE-TOFMS Equipment

All CE-TOF-MS experiments were conducted using the Agilent CE capillary electrophoresis system (Agilent Technologies, Waldbronn, Germany). LC of Agilent G1969A and G6220A Accurate-Mass TOF LC-MS systems (Agilent Technologies, Palo Alto, CA, USA) was replaced by the CE. Additionally, the Agilent 1100 and 1200 Series isocratic high-performance LC pumps, the G1603A Agilent CE-MS adapter, and the Agilent CE electrospray ionization (ESI)-MS sprayer kits (G1600AX and G7100A) were employed. For anion analysis, the Agilent G1607-60001 platinum ESI needle was used. The CE system was controlled, and data acquisition was performed using Agilent ChemStation software (versions A.10.02, B.02.01.SR1, B.03.02, and C.01.07.SE1, Agilent Technologies, Waldbronn, Germany), while system control and data acquisition utilized Agilent MassHunter software (version B.02.00, Agilent Technologies, Palo Alto, CA, USA).

### 2.4. CE-TOFMS Conditions for Cationic Metabolite Analysis

Metabolites were separated using a fused silica capillary (inner diameter 50 mm × 100 cm; Sakata Rika, Yamagata, Japan). As a pre-treatment step, the capillary was filled with 1 M formic acid (running buffer) as an electrolyte for 4 min. Approximately 5 nL of the sample solution was injected at 50 mbar for 5 s, and a voltage of 30 kV was applied. The capillary temperature was maintained at 20 °C, and the sample tray was cooled to below 4 °C. A methanol–water solution (50% *v*/*v*) containing 0.1 μM hexakis(2,2-difluoroethoxy)phosphazene was used as the sheath liquid at a 10 μL/min flow rate. Electrospray ionization (ESI)-TOFMS was performed in positive-ion mode with a capillary voltage set to 4000 V. The flow rate of the heated drying nitrogen gas (heater temperature: 300 °C) was maintained at 7 psig. For TOFMS, the fragmenter, skimmer, and octapole radiofrequency voltage (Oct RFV) were set to 75, 50, and 125 V, respectively. Automatic recalibration of each acquired spectrum was performed using reference standard substances: protonated methanol dimer isotopic ions (2MeOH + H)+, *m*/*z* 66.063061, and [hexakis(2,2-difluoroethoxy)phosphazene + H]+, *m*/*z* 622.028963. Accurate mass data were acquired at a rate of 1.5 spectra/second over the *m*/*z* range of 50–1000.

### 2.5. CE-TOFMS Conditions for Anionic Metabolite Analysis

Anion analyses were conducted using a commercially available COSMO (+) capillary (inner diameter 50 mm × 105 cm) coated with a chemically modified cationic polymer as the separation capillary. As a pre-treatment step, the capillary was filled with 50 mM of ammonium acetate (pH 3.4) for 2 min, followed by a 5 min buffer flow. Ammonium acetate solution (50 mM, pH 8.5) was used for the CE separation electrolyte solution. The sample solution (30 nL) was injected at 50 mbar for 30 s, and a voltage of 30 kV was applied. A 50% methanol–water solution containing 5 mM of ammonium acetate (*v*/*v*) with 0.1 μM of hexakis was supplied as a sheath liquid. ESI-TOFMS was performed in negative-ion mode with the capillary voltage set to 3500 V. In TOFMS, the fragmenter, skimmer, and octapole radiofrequency voltage (Oct RFV) were set to 100, 50, and 200 V, respectively. Automatic recalibration of each acquired spectrum was performed using reference standard substances: [deprotonated acetic acid dimer (2CH_3_COOH-H)]+ isotopic ion, *m*/*z* 120.038339, and [(hexakis + deprotonated acetic acid) (CH_3_COOH-H)]−, *m*/*z* 680.035541. Precise mass data were acquired at 1.5 spectra/second over the *m*/*z* range of 50–1000. Other conditions remained consistent with those used for cationic metabolite analysis.

### 2.6. LC-MS/MS Conditions for Polyamine Analysis

The LC system was an Agilent Technologies 1290 Infinity (Agilent Technologies, Santa Clara, CA, USA), equipped with a HiP sampler, quaternary pump, and column compartment. Chromatographic separation was performed at 40 °C using an ACQUITY BEH C18 column (2.1 i.d. × 50 mm, 1.7 µm; Waters, Milford, MA, USA). The mobile phase consisted of solvent A (water with 0.1% formic acid and 1.5 mM heptafluorobutyric acid) and solvent B (methanol with 1.5 mM HFBA), delivered at a flow rate of 0.4 mL/min. The gradient elution profile (mobile phase A(%)/mobile phase B(%)) was set as follows: 99:1 (0 min), 99:1 (0.6 min), 58:42 (0.8 min), 58:42 (1.8 min), 50:50 (2.3 min), 50:50 (3.0 min), 5:95 (4.0 min), and 5:95 (5.0 min).

MS detection was performed using the Agilent Technologies 6460 Triple Quadrupole Mass Spectrometer. Samples were analyzed in positive-ion mode. The instrument parameters were set as follows: drying gas temperature at 350 °C, drying gas flow rate at 13 L/min, nebulizer pressure at 55 psig, and Vcap at 3500. Specific MRM transitions, fragmentor voltages, and collision energies were optimized for each compound analyzed [19]. The systems were controlled, and raw data were collected by Agilent MassHunter Qualitative Analysis (ver. B.06.00) and Quantitative QqQ Analysis software (ver. B.07.00), including features such as MassHunter Optimizer and Dynamic Multiple Reaction Monitoring Mode (DMRM).

### 2.7. Data Analysis

Raw data from CE-TOFMS and LC-QQMS were processed using MasterHands (ver. 2.20.0.3, Keio University, Yamagata, Japan) and Agilent MassHunter Qualitative Analysis and Quantitative QqQ Analysis software (ver. 10.0 Agilent). Data processing of both software included detection of the background noise subtracted peak, peak area integration, and relative area calculation by dividing the peak area of each metabolite by the area of the internal standards [23]. The migration time of CE-MS was corrected by a dynamic programming method that tries to match corresponding peaks between samples as much as possible [24]. The name of the metabolite was estimated based on the corrected migration times and the *m*/*z* value compared with the standard compounds [25]. The isotope distributions of each peak were confirmed. A spike test was also performed.

Plasma samples were measured in four batches. The standard mixture was measured in each batch. The absolute concentration of the metabolites in the plasma samples was calculated from the standard curve data measured in the same batch. The internal standards with the same concentration were added to all standard mixtures and plasma samples. If a difference of more than 20% is observed in the internal standard size across all data, the entire batch is recalibrated and re-measured.

In addition, a quality control sample was measured in each lot to minimize unexpected bias. All metabolite peaks in the quality control sample are also monitored, and if they vary by more than 20% on average, they are re-measured after calibration and maintenance. The plasma sample data confirmed that most peaks were within the calibration curve. Peaks below the lower limit of quantitation were treated as not detected. Our protocol, which includes multiple bath measurements, was published elsewhere. The upper limit of detection, the lower limit of detection, and the recovery rate were available in these references [26,27].

Subsequent statistical analysis utilized the concentration matrix of identified metabolite concentrations. The data were randomly split into two datasets, and all statistical analyses were conducted for each dataset. Absolute metabolite concentrations (μmol/L) were used for statistical analysis. Heat maps and volcano plots were employed to visualize the overall profiles. The Mann–Whitney test was used to evaluate the difference in the metabolite concentration between the data of stage IV and stages I–III. The area under the operative characteristic (ROC) curve (AUC) was used to evaluate the discriminatory ability of metabolites between the two groups. Pathway-level differences were analyzed using Enrichment and Pathway analyses implemented in MetaboAnalyst (ver. 6.0, https://metaboanalyst.ca/ (accessed on 1 June 2024)) [28]. Kyoto encyclopedia and genes and genomes (KEGGs) pathways were used for both analyses. The pattern hunter function with the Spearman correlation option was used to identify metabolites that increased or decreased depending on the stage. The analysis used GraphPad Prism (ver. 9.5.1, GraphPad Software, San Diego, CA, USA).

## 3. Results

Patient characteristics are summarized (Table 1). The gender distribution was one female and three males, the median age was 64 (range = 47–87) years, and the median body mass index was 23.5 (17.1–31.7) kg/m^2^ in the healthy controls (n = 4). The gender distribution was 19 females and 24 males, the median age was 70 (range = 51–84) years, and the median body mass index was 21.2 (17.9–29.8) kg/m^2^ in the polyps (n = 43). We included 208 CRC patients (stages I–III 192 and stage IV 16). Gender (*p* = 0.797), age (*p* = 0.793), and body mass index (*p* = 0.387) showed no significant differences in both groups. Compared to stages I-III, stage IV tended to have a larger tumor size (*p* = 0.061).

**Figure 1:**
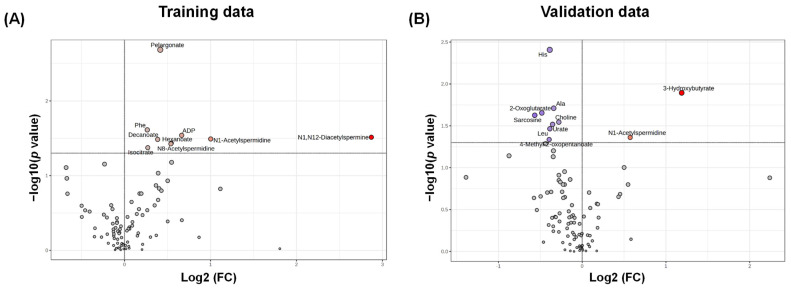
Volcano plots for the comparison of metabolome profiles between stages I–III and stage IV. (**A**) Training data. (**B**) Validation data. The X-axis represents log_2_-fold change (stage IV/stages I–III), and the Y-axis shows the −log_10_(*p*) values for each metabolite. The *p*-values were calculated using the Mann–Whitney U test. The horizontal line indicates −log_10_(*p*) = 1.3, and the metabolites above this line indicate significant differences (*p* < 0.05). Metabolites with log_10_(*p*) ≥ 1.3 are color-coded in red (higher in stage IV) or blue (higher in stages I–III).

**Figure 2:**
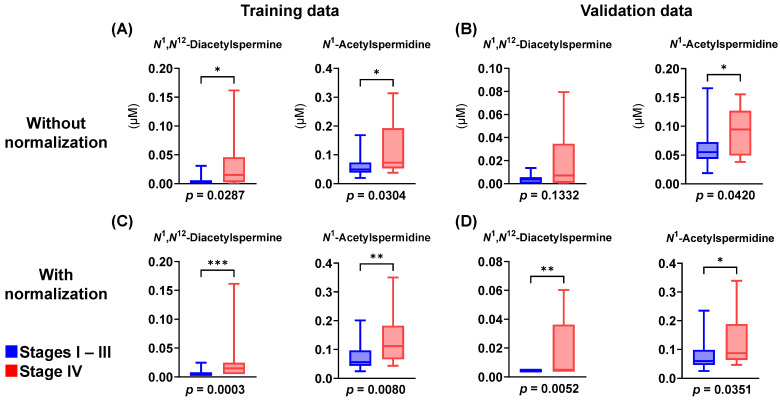
Box plots of acetylated polyamines, including *N*^1^,*N*^12^-diacetylspermine and *N*^1^-acetylspermidine. (**A**,**B**) Absolute concentration without normalization. (**C**,**D**) Relative concentration with normalization. (**A**,**C**) Training data. (**B**,**D**) Validation data. Horizontal bars of box plots indicate data of 100, 75, 50, 25, and 0%. * *p* < 0.05, ** *p* < 0.01, and *** *p* < 0.001 (Mann–Whitney test).

**Figure A1:**
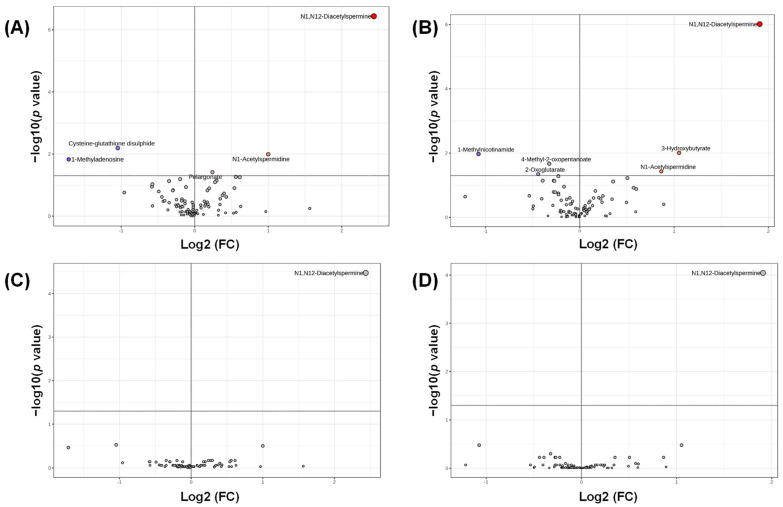
Volcano plots for comparing metabolome profiles between stages I–III and stage IV. (**A**,**C**) Training data. (**B**,**D**) Validation data. The X-axis represents log_2_-fold change (stage IV/stages I–III), and the Y-axis shows −log10(*p*) values of each metabolite. The *p*-values were calculated using the Mann–Whitney U test. The horizontal line indicates −log_10_(*p*) = 1.3, and the metabolites above this line indicate significant differences (*p* < 0.05). Metabolites with log_10_(*p*) ≥ 1.3 are color-coded in red (higher in stage IV) or blue (higher in stages I–III). (**A**,**B**) Metabolite concentrations processed with quantile normalization are used. (**C**,**D**) *p*-values are corrected by a false discovery rate.

In this study, 122 metabolites were identified and quantified using CE-TOFMS and LC-QQQMS. Among these, 94 metabolites (detected in ≥50% of the samples) were subsequently used for further analysis. The data were randomly split into two datasets: training and validation data. The patient characteristics showed no significant differences (Table A1). A comparison of stages I–III and IV data was performed. The volcano plot using absolute concentrations of each metabolite (Figure 1) illustrates the differences between stages I–III and IV data. Eight metabolites showed a significantly higher concentration in stage IV of the training data (Figure 1A). Two metabolites showed significantly higher concentrations, and eight metabolites showed significantly lower concentrations in stage IV of the validation data (Figure 1B). Training data included three acetylated polyamines, while validation data included only *N*^1^-acetylspermidine, and thus this metabolite had a consistently higher concentration in both datasets. In the training data, the metabolites with non-significant differences at Y < 1.3 were evenly distributed on both sides of the X = 0 axis. However, they were more prevalent in the validation data to the left of X < 0. Thus, we compared the data processed with quantile normalization to eliminate variations in overall metabolite concentrations (Figure 2). Two acetylated polyamines, *N*^1^-acetylspermidine and *N*^1^,*N*^12^-diacetylspermine, showed themselves to be consistently elevated in both datasets (Figure A1A,B). *N*^1^,*N*^12^-diacetylspermine showed itself to be consistently elevated even when using a false discovery rate-corrected *p*-value (Figure A1C,D).

**Figure A2:**
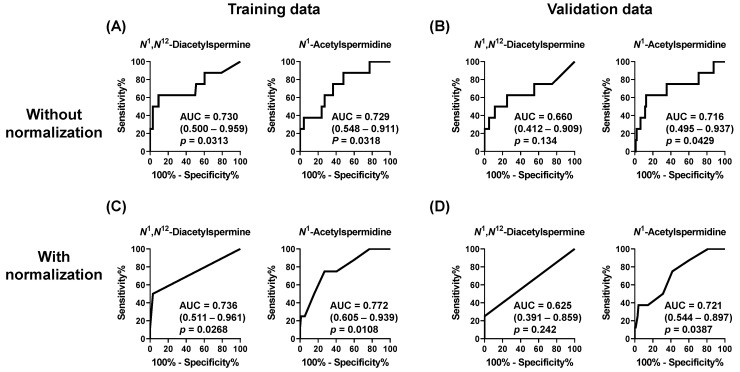
Receiver operating characteristic (ROC) curves for metabolites distinguishing stage IV data from stages I–III data. The area under the ROC curve, 95% confidence interval, and *p*-value are shown. (**A**,**B**) Absolute concentration without normalization. (**C**,**D**) Relative concentration with normalization. (**A**,**C**) Training data. (**B**,**D**) Validation data.

**Figure A3:**
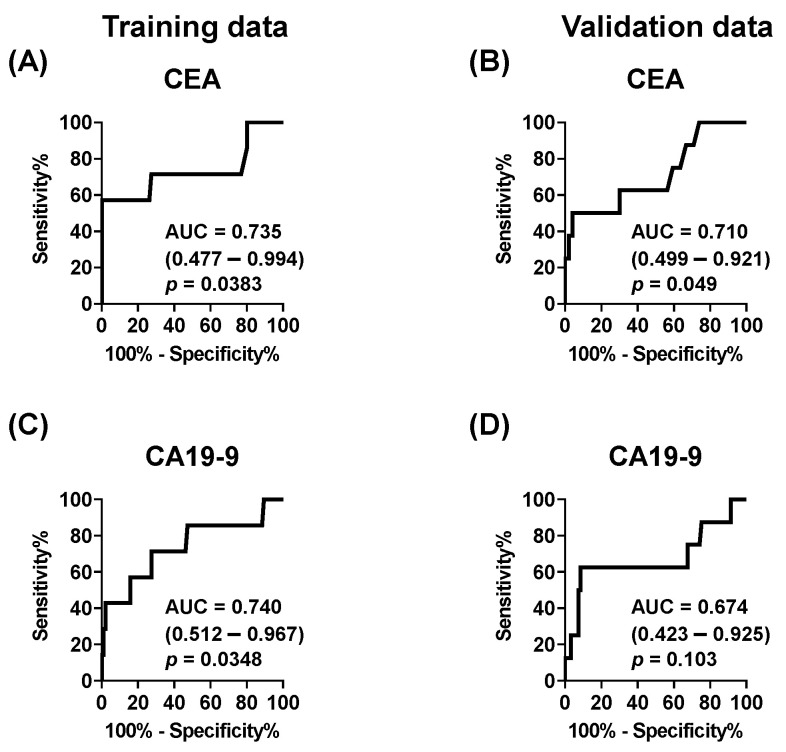
Receiver operating characteristic (ROC) curves for tumor makers distinguishing stage IV data from stages I–III data. The area under the ROC curve, 95% confidence interval, and *p*-value are shown. (**A**,**B**) CEA. (**C**,**D**) CA19-9. (**A**,**C**) Training data. (**B**,**D**) Validation data. Missing data are as follows: (**A**) stages I–III (n = 1) and stage IV (n = 1), (**B**) no, (**C**) stages I–III (n = 1) and stage IV (n = 1), and (**D**) stages I–III (n = 3).

ROC curves of *N*^1^-acetylspermidine and *N*^1^,*N*^12^-diacetylspermine are depicted to discriminate stage IV data from stages I–III data (Figure A2). Using the data without normalization, *N*^1^,*N*^12^-diacetylspermine and *N*^1^-acetylspermidine showed similar AUC values, 0.70 (95% [Confidence interval; CI]: 0.500–0.959) and 0.729 (95% CI: 0.548–0.911), respectively, in the training data (Figure A2A). These discrimination abilities were smaller in the validation data: 0.660 (95% CI: 0.412–0.909) and 0.716 (95% CI: 0.495–0.937) (Figure A2B). The data with normalization showed higher AUC values: 0.736 (95% CI: 0.511–0.961) and 0.772 (95% CI: 0.605–0.939) in the training data (Figure A2C). The data with normalization showed AUC values of 0.625 (95% CI: 0.391–0.859) and 0.721 (95% CI: 0.544–0.889) in the validation data (Figure A2D). ROC curves of tumor markers CEA and CA19-9 are depicted (Figure A3). The AUC values of CEA were 0.735 (95% CI: 0.477–0.996) and 0.710 (95% CI: 0.499–0.921) in the training and validation data, respectively. Those of CA19-9 were 0.740 (95% CI: 0.512–0.967) and 0.674 (95% CI: 0.423–0.925) in the training and validation data, respectively.

**Figure 3:**
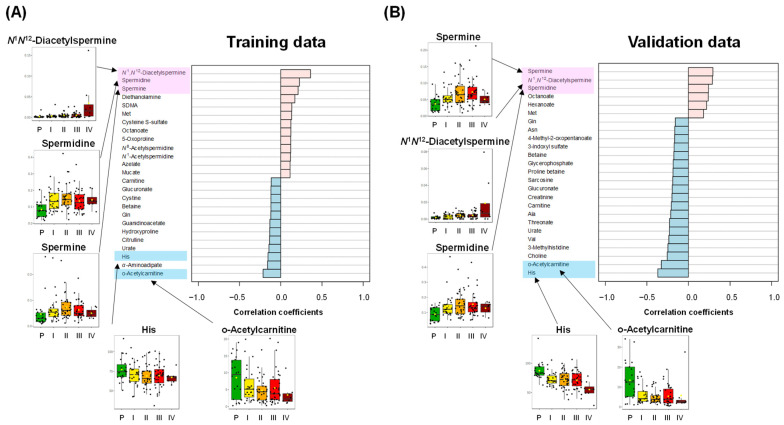
Metabolite with an increasing or decreasing trend with progress. PatternHunter function of MetaboAnalyst with Spearman correlation option is used. (**A**) Training data. (**B**) Validation data. Metabolites with positive–negative correlations are colored pink and light blue, respectively, and they ranked high in both datasets. The box plots of these metabolites are shown.

Metabolites that increase or decrease trends with cancer progression were also analyzed (Figure 3). The PatternHunter function in Metaboanalyst was used to explore the metabolites based on their correlation with cancer stages. *N*^1^,*N*^12^-diacetylspermine and two non-acetylated polyamines (spermidine and spermine) showed consistently increased trends with progression. The difference in the concentrations of *N*^1^,*N*^12^-diacetylspermine between stage IV and other data was relatively high. The spermidine and spermine concentrations increased from polyp to stage II, while these decreased or were constant in stages III and IV. Meanwhile, o-acetylcarnitine and histidine (His) showed decreasing trends. The concentration of His decreased monotonically. The concentration of o-acetylcarnitine of CRC was lower than polyp data, while the data of stage III were higher than the other stage data.

**Figure 4:**
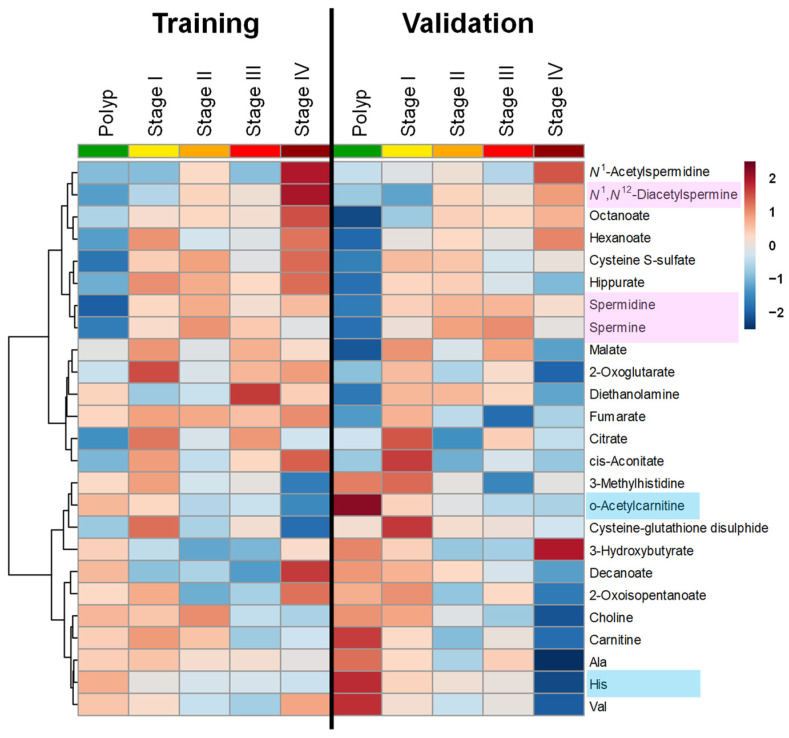
Heat map to show metabolite concentrations. Metabolites showing high variance (top 25 by ANOVA) are shown. Log_10_-transformed absolute concentration is clustered by Euclidean distance with Ward clustering methods. The metabolites with increasing and decreasing trends with progress found in Figure 3 are colored.

The heat map, including training and validation data, is shown in Figure 4. The upper metabolites, including polyamines, tended to have relatively low values in polyps and higher concentrations toward the right. Metabolites at the bottom of the figure, including amino acids, showed the opposite trend.

**Figure 5:**
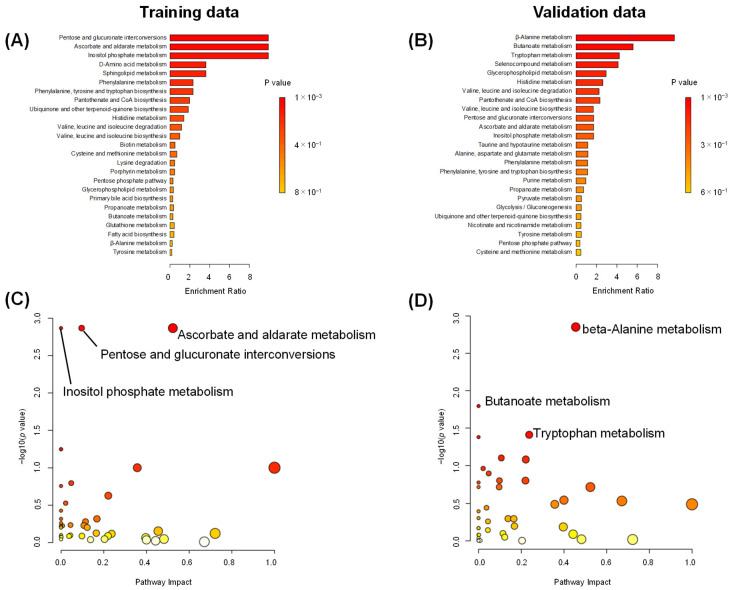
Pathway-level differences between stages I–III and stage IV. (**A**,**B**) Enrichment Analysis. The X-axis and colors represent the enrichment ratio and *p*-value for each pathway, respectively. (**C**,**D**) Pathway analysis. Both analyses use KEGGs Pathways. (**A**,**C**) Training data. (**B**,**D**) Validation data. The X- and Y-axes represent the impact score and −log_10_(*p*) for each pathway, respectively.

Pathway-level differences in stages I–III and stage IV were analyzed in the Enrichment and Pathway analysis (Figure 5). Enrichment analysis resulted in (1) pentose and glucuronate interconversion, (2) ascorbate and aldarate metabolism, and (3) inositol phosphate metabolism as highly differentiated pathways in training data (Figure 5A). This analysis resulted in (1) beta-alanine metabolism, (2) butanoate metabolism, and (3) tryptophan metabolism as highly differentiated pathways in validation data (Figure 5B). Although these top 3-ranked data showed no consistency, histidine metabolism was ranked 10th and 6th in training and validation data, respectively. Valine, leucine, and isoleucine degradation and biosynthesis were also ranked relatively high in both datasets. Pathway analyses also showed similar results (Figure 5C,D).

**Figure A4:**
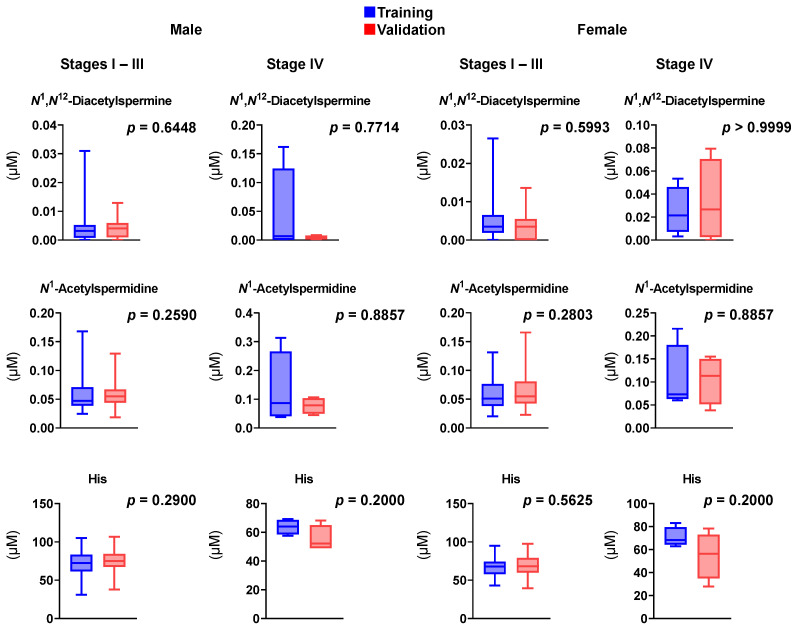
Sex-dependent differences in box plots of acetylated polyamines, including *N*^1^,*N*^12^-diacetylspermine, *N*^1^-acetylspermidine, and histidine (His). Absolute concentrations without normalization are used. Horizontal bars of box plots indicate the data of 100, 75, 50, 25, and 0%. *p* < 0.05 (Mann–Whitney test).

**Figure A5:**
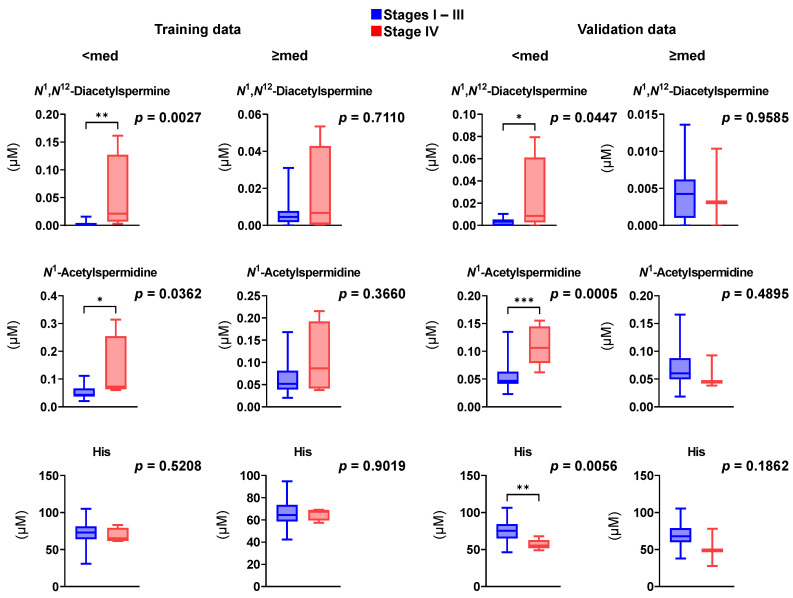
Age-dependent differences in box plots of acetylated polyamines, including *N*^1^,*N*^12^-diacetylspermine, *N*^1^-acetylspermidine, and histidine (His). The data were compared by splitting the data≥ and <the median age of 69.5. Absolute concentrations without normalization are used. Horizontal bars of box plots indicate the data of 100, 75, 50, 25, and 0%. * *p* < 0.05, ** *p* < 0.01, and *** *p* < 0.001 (Mann–Whitney test).

The metabolite concentration of *N*^1^,*N*^12^-diacetylspermine, *N*^1^-acetylspermidine, and His is listed in Table A2. Relationships between sex and three metabolites are shown in Figure A4. There were no significant differences between male and female data regarding these three metabolites. Age-dependent differences in these three metabolites are shown in Figure A5. *N*^1^,*N*^12^-diacetylspermine and *N*^1^-acetylspermidine showed significant differences in the younger data (<median of the age, 69.5), while these metabolites showed no significant differences in the older data.

## 4. Discussion

In this study, we investigated changes in the metabolomic profile of plasma samples collected from CRC patients using non-targeted metabolome analysis. The concentrations of acetylated polyamines such as *N*^1^,*N*^12^-diacetylspermine and *N*^1^-acetylspermidine were elevated in stage IV data compared to the other stages. *N*^1^,*N*^12^-diacetylspermine, spermine, and spermidine showed an increasing trend along with progress. In previous studies, the concentration of acetylated polyamines in the urine and saliva of CRC patients was higher than in the non-cancer group [18,19]. Furthermore, elevated concentrations of *N*^1^,*N*^12^-diacetylspermine in serum or plasma have been observed in patients with lung and breast cancer in comparison to the non-cancer group [29,30].

In normal cells, the tumor suppressor adenomatous polyposis coli (APC) regulates the transcription of the oncogene MYC and controls the degradation of ornithine decarboxylase (ODC) by ornithine decarboxylase antizyme (OAZ). However, in CRC patients, mutated or deleted APC leads to decreased OAZ levels and reduced MYC inhibition, resulting in increased expression of the ODC gene. Consequently, polyamine synthesis becomes activated. The activation of spermine/spermidine *N*^1^-acetyltransferase (SSAT) leads to an abundance of acetylated polyamines. These acetylated polyamines also spread to the surrounding tissues and blood vessels [31]. The high concentration of acetylated polyamines in colorectal cells has been consistently observed [32,33]. The depletion of polyamines by SSAT significantly inhibited cell proliferation, migration, and invasion through the AKT/GSK3β/β-catenin pathway in CRC cells [34]. SSAT expression levels are associated with tumor grade and stage [35], consistent with our polyamine profile observations.

In this study, we consistently confirmed that the concentrations of these metabolites in plasma samples from stage IV CRC patients were elevated. On the other hand, histidine was significantly lower in the data from stages I–III. A reduction in histidine and tryptophan levels in plasma samples has been reported in various cancers using amino acid profiling [36,37]. Our data observed a gradual decrease in histidine concentration as the stage progressed. Although tryptophan levels also decreased with disease progression, no significant difference existed between the data from stages I to III and stage IV. A metabolomic study indicated that the concentrations of nine amino acids, including alanine and aspartic acid, differed in CRC patients with and without lymph node metastasis [38]. However, our findings did not align with these results. A study investigating metabolic differences in CRC tissues with and without lymph node metastasis reported alterations in glycolysis, glutaminolysis, fatty acid metabolism, choline metabolism, and amino acids [39]. Polyamines were not detected by their measurement protocols.

Comparative studies on non-metabolite biomarkers across different CRC stages have been reported, such as CTC, circulating free DNA (cfDNA), circulating tumor DNA (ctDNA), and microRNAs (miRNAs). For example, a quantitative correlation between CTCs and CRC stage was reported [40]. Notably, CTCs have shown higher positive detection rates than the conventional serum marker CA19-9, especially in stage II and III CRC patients [41]. Various studies of cfDNA and ctDNA have demonstrated that higher levels of DNA alterations correlate with shorter overall survival in metastatic CRC patients [42,43,44]. In studies targeting stages I–III, ctDNA has effectively identified recurrence after curative treatment, independently of other clinical risk factors [45].

Our metabolomic analysis comprehensively investigated metabolites rather than tumor cells like CTCs. We found that acetylated polyamines were high in stage IV. These metabolites were repeatedly reported as potential biomarkers to discriminate CRC from healthy controls. We also found that several other metabolites, such as His, showed decreasing trends with progress. Metabolomic aberrancies in CRC may play a role in inhibiting tumor recurrence and improving prognosis [46].

Several future perspectives emerge from our study, particularly regarding the correlation between commonly used CRC markers, CEA and CA19-9, and plasma metabolomics. Prior research suggested a limited correlation between these tumor markers and urinary metabolites [21]. However, exploring their relationship in plasma is essential, considering their potential complementary roles. We identified specific biomarkers that significantly increase in stage IV compared to stages I–III, which could have a substantial clinical impact. Large-scale prospective trials combining these markers with other biomarkers are desirable.

Our study has limitations. This research was conducted as a single-center retrospective study. There are a few cases of stage IV, especially. This issue may result in a non-significant difference using the FDR-corrected *p*-value when comparing stages I–III data to stage IV data. The staging ability of this metabolite should be validated in larger cohorts. LC-MS/MS has identified polyamines based on MS fragments. However, other metabolite names assigned to the CE-TOFMS data were of lower confidence. This problem was caused by the fact that MS fragments were not available. MS/MS should be used to identify more confidently. Future research should compare metabolites in other cancer types.

## 5. Conclusions

In conclusion, *N*^1^,*N*^12^-diacetylspermine, known as a CRC detection agent, showed potential to discriminate stage IV from other stages. In addition, we could identify stage-specific changes in other comprehensively analyzed metabolic profiles.

## Figures and Tables

**Table 1:** Patient characteristics.

Feature		Controls	Polyps	Stages I–III	Stage IV	*p*-Value ^d^
Gender	Female	1	19	87	8	0.7972 ^a^
	Male	3	24	105	8	
Age	Median	64	70	70	69	0.793 ^b^
	Min	47	51	27	53	
	Max	87	84	97	84	
Body mass index (kg/m^2^)	Median	23.5	21.2	22.1	21.4	0.387 ^b^
	Min	17.1	17.9	14.9	13.7	
	Max	31.7	29.8	33.7	31.8	
Tumor size (mm)	Median	N/A ^c^	N/A ^c^	39	57	0.061
	Min	N/A ^c^	N/A ^c^	7	23	
	Max	N/A ^c^	N/A ^c^	125	176	

^a^ Fisher’s exact test. ^b^ Mann–Whitney test. ^c^ Not applicable. ^d^ *p*-value for the comparison between stages I–III and stage IV.

## Data Availability

Data are available upon request.

## Appendix A

**Table A1:** Patient characteristics in training and validation datasets.

Characteristics		Training	Validation	*p*-Value
Age (Mean ± SD)		68.07 ± 11.88	68.23 ± 11.83	0.951 ^a^
Age (<med/≥med)	Stages I–III	51/45	44/52	0.312 ^b^
	Stage IV	4/4	5/3	0.614 ^b^
Sex (M/F)		75/53	70/55	0.757 ^b^
	Stages I–III	54/42	51/45	0.664 ^b^
	Stage IV	4/4	4/4	>0.9999 ^b^
Stage (n)				
	Polyp	22	21	0.9997 ^b^ (0.9987) ^c^
	I	27	27	
	II	33	34	
	III	36	35	
	IV	8	8	

^a^ Mann–Whitney test. ^b^ Chi-square test. ^c^ Chi-square test using only CRC data.

**Table A2:** Metabolite concentration (μmol/L).

Metabolites	Stage	Training	Validation	*p*-Value ^a^
*N*^1^,*N*^12^-Diacetylspermine	Polyp	0.00197 ± 0.00383	0.00183 ± 0.00168	0.292
	Stage I–III	0.00467 ± 0.00551	0.00393 ± 0.00334	0.982
	Stage IV	0.0344 ± 0.0543	0.0187 ± 0.00999	0.504
*N*^1^-Acetylspermidine	Polyp	0.0545 ± 0.0345	0.0588 ± 0.024	0.272
	Stages I–III	0.00467 ± 0.00551	0.00393 ± 0.00334	0.982
	Stage IV	0.118 ± 0.0972	0.0912 ± 0.0414	>0.9999
His	Polyp	77.24 ± 15.19	87.44 ± 15.59	0.0101
	Stages I–III	69.62 ± 14.31	71.99 ± 13.97	0.233
	Stage IV	67.12 ± 7.587	55.05 ± 14.75	0.0379

^a^ Mann–Whitney test.
